# Evaluation of Accuracy of Episiotomy Incision in a Governmental Maternity Unit in Palestine: An Observational Study

**DOI:** 10.1155/2018/6345497

**Published:** 2018-10-29

**Authors:** Hadil Y. Ali-Masri, Sahar J. Hassan, Kaled M. Zimmo, Mohammed W. Zimmo, Khaled M. K. Ismail, Erik Fosse, Hasan Alsalman, Åse Vikanes, Katariina Laine

**Affiliations:** ^1^Department of Obstetrics, Palestine Medical Complex, Ramallah, State of Palestine; ^2^The Intervention Centre, Oslo University Hospital, Rikshospitalet, 4950 Nydalen, 0424 Oslo, Norway; ^3^Institute of Clinical Medicine, Faculty of Medicine, University of Oslo, 1171 Blindern, 0318 Oslo, Norway; ^4^Faculty of Pharmacy, Nursing and Health Professions, Birzeit University, Birzeit Box14, Ramallah, State of Palestine; ^5^Department of Obstetrics, Al Aqsa Martyrs Hospital, Gaza, State of Palestine; ^6^Department of Obstetrics, Al Shifa Hospital, Gaza, State of Palestine; ^7^Department of Obstetrics and Gynaecology, Faculty of Medicine, Ain Shams University, Cairo, Egypt; ^8^Department of Obstetrics, Oslo University Hospital, Ullevål, 0424 Oslo, Norway; ^9^Department of Health Management and Health Economics, Institute for Health and Society, University of Oslo, Oslo, Norway

## Abstract

Episiotomy should be cut at certain internationally set criteria to minimize risk of obstetric anal sphincter injuries (OASIS) and anal incontinence. The aim of this study was to assess the accuracy of cutting right mediolateral episiotomy (RMLE). An institution-based prospective cohort study was undertaken in a Palestinian maternity unit from February 1, to December 31, 2016. Women having vaginal birth at gestational weeks ≥24 or birthweight ≥1000 g and with intended RMLE were eligible (*n*=240). Transparent plastic films were used to trace sutured episiotomy in relation to the midline within 24-hour postpartum. These were used to measure incisions' distance from midline, and suture angles were used to classify the incisions into RMLE, lateral, and midline episiotomy groups. Clinical characteristics and association with OASIS were compared between episiotomy groups. A subanalysis by profession (midwife or trainee doctor) was done. Less than 30% were RMLE of which 59% had a suture angle of <40° (equivalent to an incision angle of <60°). There was a trend of higher OASIS rate, but not statistically significant, in the midline (16%, OR: 1.7, CI: 0.61–4.5) and unclassified groups (16.5%, OR: 1.8, CI: 0.8–4.3) than RMLE and lateral groups (10%). No significant differences were observed between episiotomies cut by doctors and midwives. Most of the assessed episiotomies lacked the agreed criteria for RMLE and had less than optimal incision angle which increases risk of severe complications. A well-structured training program on how to cut episiotomy is recommended.

## 1. Introduction

Episiotomy is a surgical incision of the perineum used to facilitate childbirth [1]. Episiotomy may cause bleeding, infection, dyspareunia, and postpartum pain [1] and should not be used routinely but only when indicated [2]. The use of episiotomy has declined markedly from 60% in 1979 to 9.4% in 2011 in the United States [3, 4] and from 20% in 1975 to 7% in 2010 in some Scandinavian countries [5, 6]. The rate in low-income countries remains relatively high. At least 75% of primiparous women in Jordan and Yemen receive episiotomy as a routine [7, 8].

Different episiotomy types have been described. Mediolateral and midline episiotomies are the most commonly used incisions [9]. In 2008, Kalis et al. [10] elaborated discrepancies in the definition of mediolateral episiotomy in their survey among 122 hospitals across Europe. They reported that approximately 50% of the surveyed hospitals had incomplete or no definition of mediolateral episiotomy. Subsequently, Kalis et al. [9] suggested classification criteria for different types of episiotomy techniques to be used as reference for future research.

Midline episiotomies are associated with an increased risk of obstetric anal sphincter injuries (OASIS), which represent the most determinant factor for female anal incontinence [11, 12]. However, suboptimal characteristics of mediolateral episiotomy such as short- and narrow-angled incisions have also been found to increase OASIS risk [13–16]. Hence, the National Institute of Clinical Excellence guidelines recommend using the mediolateral technique, if an episiotomy is indicated, at an angle of 45°–60° from the vertical axis [17].

In Palestine, right mediolateral episiotomy (RMLE) is the standard used technique. Midwives are allowed to cut episiotomy only in some hospitals, depending on the institution's internal protocol and the individual's experience. According to the Palestinian Central Bureau of Statistics [18], the episiotomy rate in 2004 for West Bank and Gaza was reported to be 10.4% and 15%, respectively. In 2005, Wick et al. [19] published a study comparing routine practices of normal childbirth in eight Palestinian maternity units with the evidence-based guidelines and reported that six of eight units used routine episiotomy for primiparous women. Indeed, a more recent study by Zimmo et al. [20], including six governmental maternity units in Palestine, has shown an overall episiotomy rate of 28.7% and 78.8% among women having their first vaginal birth.

In view of the above, this study was conducted to assess the quality of what could be the most commonly performed intrapartum surgical intervention in Palestine, an episiotomy.

The main aim of this study was to evaluate the accuracy and characteristics of RMLE cut by doctors and midwives in relation to the international recommendations.

## 2. Materials and Methods

Reporting of this prospective cohort study followed the strengthening the reporting of observational studies in epidemiology (STROBE statement version 4, 2007) guidelines. The study was conducted between February 1, 2016 and December 31, 2016 in a teaching maternity unit in the West Bank (Palestine) which has 4000–5000 births per annum. Midwives attend low-risk spontaneous vaginal births while doctors are involved in high-risk spontaneous and operative vaginal births. If indicated, RMLE is the only recommended and used technique. Almost all doctors who cut episiotomies are trainee doctors, but only experienced midwives are allowed to cut episiotomy.

Women with episiotomy who had spontaneous or operative vaginal births at ≥24 gestational weeks or ≥1000 gm in birthweight were eligible for inclusion. The principle investigator (H. Y. AM) and two assistants (trainee doctors) recruited women, immediately or within 24-hour postpartum, almost on daily basis during the morning shift and on evening and night shifts when the two assistants were on call. The study purpose and procedure were explained to potential participants. Two hundred and forty-eight women were invited, and all agreed to participate and provided verbal consent. The methodology used in this study followed the method that was formerly used by Fodstad et al. [21]. The principle investigator and the two assistants assessed the episiotomy incision immediately after suturing or the next day before a woman was sent home. Women were placed in the lithotomy position, resembling their position at birth, legs flexed at hip joints with buttocks and feet resting on the edge of the bed to achieve full visualization of the perineum. A transparent plastic film was placed on the perineum, and a permanent marker pen was used to mark the midline, identified as a line from midpoint of the introitus downward through the anal orifice. The sutured episiotomy incision was then drawn and marked as suture line. The posterior fourchette and the vaginal and anal orifices were marked on the plastic film. Afterwards, the drawn episiotomy incisions' characteristics were measured by the principle investigator, and a random set of these measurements were checked by the two authors (K. Z. and M. Z.) using the same measuring technique. The episiotomy incision length and distance from midposterior fourchette (distance from midline) were measured in millimeters using a ruler, and the episiotomy suture angle from the vertical axis was measured in degrees with a protractor. Based on Kalis et al.'s [10] classification criteria, measurements of distance from midline and the suture angle were used to classify the episiotomy incisions into four groups: RMLE, lateral, midline, and unclassified episiotomy. The suture angle categorization was based on Kalis et al.'s [22] demonstration that the suture angle is 15°–20° less than the incision angle. Accordingly, an episiotomy incision angle of 40°–60° was considered equivalent to a suture angle of 25°–40°, respectively.  Table 1 presents the classification criteria.

Studies consistently reported that a mediolateral episiotomy incision angle of 60° or more is associated with less frequency of OASIS [14, 16, 22]. Therefore, we performed a subanalysis using a cutoff suture angle for an optimum RMLE of ≥40° (i.e., equivalent to incision angle of ≥60°).

This study was part of a larger multicenter study on obstetric perineal trauma and birth complications in Palestine. Birth attendants collected data using special case registration forms which were subsequently entered into similar electronic forms [23]. Each woman had a serial number on the paper form matching that on the electronic form. The episiotomy drawings were given the same serial numbers on the data collection forms to enable retrieving the participants' anonymous data.

### 2.1. Ethical Approval

The Palestinian health research council (Reference no.: BHRC\HC\13\15), the Regional Committee for Medical and Health Research Ethics in South Eastern Norway (REK 2014/1727), and the Norwegian Data Inspectorate (17/00082-2/GRA) approved this study as health quality research which can be implemented without its approval within the ordinary health services arrangements as long it is done in accordance with common rules for health care services in Palestine and Norway regarding confidentiality and privacy. Verbal consent was considered valid.

### 2.2. Statistical Analysis

SPSS version 24.0 (IBM, Armonk, NY, USA) was used for statistical analysis. The study population characteristics were explored using descriptive analyses. To avoid small accounts within each episiotomy group, RMLE and lateral episiotomies were combined to form the RMLE-lateral group. Subsequently, differences in clinical characteristics were assessed between the different episiotomy groups using Fisher's exact test for categorical and one-way ANOVA test for continuous variables. Logistic regression analysis was used to assess the association between OASIS and each episiotomy group. A subanalysis according to the profession of the person who cut the episiotomy (midwife or trainee doctor) was also done. Differences between trainee doctors and midwives with regard to episiotomy type and characteristics were assessed using Fisher's exact test for categorical and independent *t*-test for continuous variables. Linear regression analysis was used to explore the association between the suture angle and distance from midline. The level of significance was set at *p* value <0.05.

## 3. Results

Two hundred forty (96.8%) women were included in the analysis after excluding eight (3.2%) due to missing data.  Table 2 shows the clinical characteristics of the study population. The episiotomy incision length ranged from 13 mm to 51 mm (mean = 27.6 mm), distance from midline from 0 mm to 27 mm (mean = 5.1 mm), and suture angle from 4° to 78° (mean = 31.7°). One hundred sixty-eight (70.0%) of episiotomy suture angles were <40°.

A significant association was found between the distance from midline and the suture angle; the longer the distance, the larger the episiotomy angle (beta coefficient 0.51, CI: 0.17–0.84, *p*=0.004).

According to the classification criteria in Table 1, 71 (29.6%) incisions were classified as RMLE. The remaining 169 (70.4%) incisions were classified as lateral in 28 (11.7%), midline in 50 (20.8%), and unclassified in 91 (37.9%).

Within the RMLE group, suture angles were <40° in 42 (59.2%) of women.

There were no significant differences in clinical characteristics of the study population by type of episiotomy (Table 3).

Thirty-three (13.8%) women sustained OASIS of which 10 (10.1%) were identified in the RMLE-lateral group, 8 (16%) in the midline episiotomy group, and 15 (16.5%) in the unclassified episiotomy group. After adjusting for parity, gestational age, birthweight, and operative birth using logistic regression analysis, the unclassified and midline episiotomy groups had 1.8 and 1.7 times more odds for OASIS than RMLE-lateral group. However, this was not statistically significant (aOR: 1.8, CI: 0.8–4.3, *p*=0.17 and aOR: 1.7, CI: 0.61–4.5, *p*=0.32, respectively). Using the same logistic regression model, birthweight and operative vaginal birth were not associated with increased odds for OASIS.

In this cohort, doctors have cut 210 (87.5%) while midwives have cut 30 (12.5%) episiotomies. Of these, 61 (29.0%) and 10 (33.3%) fulfilled RMLE criteria, respectively. The most frequent incisions cut by both doctors and midwives were of the unclassified type: 79 (37.6%) by doctors and 12 (40.0%) by midwives. No significant variations were detected between doctors and midwives with regard to frequency of episiotomy type or the incision characteristics (Table 4). However, episiotomies cut by midwives were more likely to have suture angles ≥40° than doctors (12 (40.0%) and 60 (28.6%), respectively); nonetheless, this was not statistically significant.

## 4. Discussion

This study has shown that less than 30% of episiotomies, cut with the intention of being RMLEs, fulfilled the criteria for such technique while the majority of incisions were unclassifiable. Lateral and midline episiotomies were inadvertently cut in more than 32% of women. Moreover, less than half of the RMLEs had a suture angle of ≥40° (equivalent to an estimated optimal incision angle of ≥60°). Accordingly, only 12% of all the assessed incisions were considered optimum RMLEs. These findings confirm what has been described in studies from different settings and countries. In the UK (where RMLE is supposed to be the standard technique), Andrews et al. [24] examined 98 episiotomies immediately after repair and reported that 13% were true RMLE while most incisions were closer to the midline. However, their classification was based on the definition that RMLE should be cut at an angle at least 40° from midline without specifying the incision point. Fodstad et al. [21], on the other hand, used the same classification criteria of this study to assess 300 episiotomies in Norway (where lateral episiotomy is supposed to be the standard technique) and found that 44% of the episiotomies were actually lateral while 36% were unclassifiable.

Andrews et al. [24] have also indicated that doctors have cut longer and more angled episiotomies than midwives. Similar findings have been reported by Tincello et al. [13] after evaluating episiotomies drawn on pictorial questionnaires by doctors and midwives within the same organization. In our study, we found that although episiotomies cut by midwives and doctors were fairly similar, midwives were more likely to have suture angles at ≥40°. However, it is important to highlight that all the doctors in this study were trainees.

Although differences in OASIS rates amongst the episiotomy groups in this study were statistically insignificant, midline and unclassified incisions were associated with higher rates of trauma involving the anorectal complex. Despite the small mean birthweight and low rate of operative vaginal birth, the overall OASIS rate in this cohort is quite high which could be a result of a suboptimal incision angle. Seventy percent of all the incisions and more than half of RMLE had suture angles <40° which signifies that the majority of episiotomies is cut at angles <60°. There is evidence that the risk of OASIS is progressively increasing with smaller episiotomy angles. Eogan et al. [14] indicated that, for each degree reduction in the repaired episiotomy angle, there was approximately 10% increased risk for third-degree tear. Interestingly, episiotomies with too wide suture angles were also found to be associated with increased risk of OASIS [15]. Taking into consideration that the episiotomy angle becomes smaller after the baby is born, an incision angle of 60° was found optimal [14, 16]. However, it seems that there are challenges to cutting an episiotomy at an optimal angle. In an observational study by Naidu et al. [25], 106 doctors and midwives were asked to cut mediolateral episiotomy at 60° on episiotomy incision pads. Thirty-six percent of the participants cut the episiotomy between 55° and 65°, 44% underestimated the angle at ≤55°, and 18% overestimated the angle at ≥66°. In view of that, the cutting instrument Episcissor-60 (Medivent Limited, Romsey, Hampshire, SO51 8BW, UK) was devised to help clinicians cut episiotomy at a fixed angle of 60°. When used, the Episcissor-60 has consistently achieved the recommended episiotomy angle which is supposed to reduce OASIS risk [26]. Nevertheless, the Episcissor-60 is relatively expensive and unaffordable by resource-constrained settings.

Guidelines for the optimal RMLE technique are readily available. However, we believe that the real gap is in the lack of a structured training on how to cut an episiotomy. An audit of training in obstetric perineal trauma among 150 doctors and midwives demonstrated that only 20% of doctors and 50% of midwives were satisfied with their training and many doctors reported that they have never received formal training in how to cut episiotomy [27]. Doctors and midwives in Palestine learn cutting an episiotomy solely by observation. Up-to-date and evidence-based theoretical background is often lacking, and the skill is barely practiced on simulators beforehand. Student midwives are required to learn to cut and repair episiotomy during their clinical training. Yet, achieving and maintaining competence in the procedure is difficult because the procedure is less likely to be used in multiparous low-risk births, which form the main case load in midwifery. However, an optimal training program is still undefined. Silf et al. [28] reported that the available training workshops in the UK focus on episiotomy repair but not on how to cut correct episiotomy. In addition to adherence to evidence-based guidelines and simulation training workshops, we believe that supervised bedside clinical training is also needed to improve episiotomy incisions' quality.

The main strength of our study is that it is the first study evaluating real-life episiotomies in Palestine conducted by a multidisciplinary team of international experts in the field. However, we recognize that there are some limitations; firstly, we were not certain about the birth position of some women whose episiotomy incisions were drawn the next day of birth. This is important because changing the flexion angle at hip can, potentially, affect the measurements. However, almost all women give birth in this unit in a lithotomy position with feet resting on the bed, not in stirrups, and therefore, we positioned the participants similarly. Secondly, one trainee doctor of the assessors was involved in cutting some of the examined episiotomies. This might have increased the correct incisions' number but not the main study conclusion. Lastly, the relatively small numbers per each episiotomy group may have underpowered the study to demonstrate a significant difference for some of our associations, such as variations in OASIS rate. Similarly, the small number of episiotomies cut by midwives, compared to doctors, may have underestimated variations in clinicians' practice. Power sample calculations not performed as this study were mainly designed to highlight the gap of evidence implementation into practice with regard to cutting RMLE and the potential detrimental consequences of this.

## 5. Conclusion

The majority of intended RMLE in this study was inaccurate and did not meet the recommended criteria. Innovative and efficient programs and flexible policies that allow regular, well-supervised, and structured training in how to cut episiotomy, as a mandatory component in doctor residency and midwifery-training programs, are needed to improve trainee doctors' and trainee midwives' competencies.

## Figures and Tables

**Table 1:** Criteria used to classify the study episiotomy incisions into four episiotomy groups.

Characteristics	RMLE	Lateral	Midline	Unclassified
Distance from midline (mm)	0–3	≥10	0–3	4–9
Suture angle (degrees)	25°–60°	25°–60°	0°–25°	All angles

**Table 2:** Clinical characteristics of the study population (*n*=240).

Characteristics	
Primiparous women	227 (94.6)
Parous women	13 (5.4)
Age	23 ± 3.8
Gestational age	39 ± 1.6
Spontaneous vaginal birth	231 (96.3)
Operative vaginal birth	9 (3.8)
OASIS	33 (13.8)
Birthweight	3157 ± 453
Length of episiotomy	27.6 ± 7.2
Distance from midline	5.1 ± 5.1
Episiotomy suture angle	31.7 ± 14.0

Categorical variables are presented in *n*/240 (%), and continuous variables are presented in mean ± SD. OASIS: obstetric anal sphincter injuries.

**Table 3:** Comparison with the clinical characteristics between different episiotomy groups. The RMLE and lateral episiotomies are combined in one group (RMLE-lateral group).

Characteristics	RML-lateral (*n*=99)	Midline (*n*=50)	Unclassified (*n*=91)	^a^ *p*
Primiparous women	95 (96.0)	48 (96.0)	84 (92.3)	0.539
Parous women	4 (4.0)	2 (4.0)	7 (7.7)	0.539
Spontaneous vaginal birth	96 (97.0)	48 (96.0)	87 (95.6)	0.910
Operative vaginal birth	3 (3.0)	2 (4.0)	4 (4.4)	0.910
OASIS	10 (10.1)	8 (16.0)	15 (16.5)	0.376
Birthweight	3147 ± 440	3236 ± 511	3130 ± 422	0.379
Length of scar	27.5 ± 7.3	29.1 ± 7.0	26.8 ± 7.0	0.187

^a^Fisher's exact test was used for categorical variables and one-way ANOVA for continuous variables. OASIS: obstetric anal sphincter injuries.

**Table 4:** Clinical characteristics of episiotomies cut by trainee doctors and midwives.

Characteristics	Trainee doctors (*n*=210)	Midwives (*n*=30)	^a^ *p* ± (95% CI)
Type of episiotomy			
RMLE	61 (29.0)	10 (33.3)	0.67
Lateral	26 (12.4)	2 (6.7)	0.55
Midline	44 (21.0)	6 (20.0)	>0.99
Unclassified	79 (37.6)	12 (40.0)	0.84
Length of episiotomy	2.8 ± 0.7	2.7 ± 0.8	0.92 (CI: −2.6–3.0)
Suture angle of episiotomy	31.8 ± 13.8	31.3 ± 15.5	0.87 (CI: −5.0–6.0)
Distance from midline	0.51 ± 0.51	0.51 ± 0.57	0.98 (CI: −2.0–2.0)
OASIS	27 (12.9)	6 (20.0)	0.20 (CI: 0.7–5.2)

Categorical variables are presented by *n* (%) and continuous variables by mean ± SD. ^a^Fisher's exact test was used for categorical variables (presented by *p* value) and independent *t*-test for continuous variables (presented by *p* value and 95% confidence interval). OASIS: obstetric anal sphincter injuries.

## Data Availability

The dataset generated and analyzed during the current study is not publicly available. Based on the recommendations of the data inspectorate, the dataset of this study is stored in a secure platform (service for sensitive data (TSD)) owned by the University of Oslo. TSD facilities are developed and operated by TSD service group at the IT Department of the University of Oslo (tsd-drift@usit.uio.no). Access to this platform requires special permission given to researchers working at the university and in other public research institutions to collect, store, analyze, and share sensitive data in compliance with the Norwegian regulations regarding individuals' privacy.
